# Risk assessment for foot ulcers among Tunisian subjects with diabetes: a cross sectional outpatient study

**DOI:** 10.1186/s12902-020-00608-2

**Published:** 2020-08-24

**Authors:** B. Zantour, S. Bouchareb, Z. El Ati, F. Boubaker, W. Alaya, W. Kossomtini, M. H. Sfar

**Affiliations:** 1 Department of endocrinology and internal medicine, Tahar Sfar hospital, 5100 Mahdia, Tunisia; 2 Department of physical medicine and rehabilitation, Tahar Sfar Hospital, 5100 Mahdia, Tunisia

**Keywords:** Diabetic foot ulcer, Risk factors, IWGDF classification, Screening

## Abstract

**Background:**

Diabetic foot is an underestimated and redoubtable diabetes complication. The aims of our study were to assess diabetic foot ulcer risk factors according to International Working Group on the Diabetic Foot (IWGDF) classification, stratify patients into risk categories and identify factors associated with higher-risk grade.

**Methods:**

Cross-sectional setting over a period of 07 months, patients were randomly selected from the diabetic outpatients attending our unit of diabetology. Questionnaire and clinical examination were made by the same physician. Patients free of active foot ulcer were included.

**Results:**

Among 230 patients evaluated, 10 had an active foot ulcer and were excluded. Five patients (2.27%) had a history of foot ulcer and 3(1.36%) had a lower-limb amputation. Sensory neuropathy, as measured by the 5.07(10 g) Semmes-Weinstein monofilament testing, was present in 23.63% of patients, whereas 36.82% had a peripheral arterial disease based on clinical findings, and 43.63% had foot deformities. According to the IWGDF classification, Group 0: 72.72%, Group 1: 5.9%, Group 2: 17.73% and Group 3: 3.63%. After univariate analysis, patients in higher–risk groups were significantly more often female, had higher age and BMI, longer diabetes duration, elevated waist circumference, low school level, retinopathy and hyperkeratosis. Multivariate logistic regression analysis identified 3 significant independent factors associated with high-risk groups: retinopathy (OR = 2.529, CI95 [1.131–5.655], *p* = 0.024), hyperkeratosis (OR = 2.658, CI95 [1.222–5.783], *p* = 0.014) and school level (OR = 0.489, CI95 [0.253–9.44], *p* = 0.033).

**Conclusions:**

Risk factors for foot ulceration were rather common in outpatients with diabetes. The screening of patients at risk for foot ulceration should start early, integrated with sustainable patient education.

## Background

Diabetic foot ulceration is one of the most severe complications of diabetes. Eighty-five percent of non-traumatic amputations in diabetic patients are preceded by foot injury [1]. The main mechanisms causing the lesions are the peripheral sensory neuropathy (PSN), the peripheral artery disease (PAD), which can be associated in varying degrees, in combination with other factors such as microvascular disease, biomechanical abnormalities and limited joint mobility [2].

It is important to identify people with diabetes at risk of developing foot lesions using a classification easy to apply in daily practice in order to facilitate diabetic foot assessment and to prioritize high-risk patients for prevention services. Several risk classification schemes have been developed [3–5]. The classification developed by the International Working Group on the Diabetic Foot (IWGDF) in 1999 [3] has been used in different studies [6–8]. Effectiveness of this classification system to predict diabetic foot complications has been demonstrated [6]. This classification is based on practical and simple clinical data. The presence of PSN, PAD, deformity and history of ulceration and / or amputation are the components of this classification [3]. In addition to these major contributing factors to foot ulcers, several factors have been demonstrated to be associated with higher risk groups [7].

Data on the prevalence of people with diabetes at risk for foot ulceration are missing in Tunisia and are rarely reported in outpatients with diabetes [8]. Thus, we aimed in this study to assess diabetic foot ulcer risk factors according to the IWGDF classification, stratify patients into risk categories and identify factors associated with higher-risk grade in people with diabetes in a tunisian diabetes-endocrinology outpatient department.

## Methods

### Study design

We conducted a cross-sectional study to determine the prevalence of risk factors of foot ulceration (PSN, PAD, deformity and history of ulceration and / or amputation) and then classify the patients according to the IWGDF classification system. We performed an univariate then a multivariate analysis to identify significant factors associated with higher foot ulceration risk.

### Study population

Patients were randomly selected from the patients attending the diabetes-endocrinology outpatient department in Tahar Sfar hospital in Mahdia over a period of 07 months. The same physician made the interrogatory, the clinical examination and collected complementary exams data from all selected patients at the outpatient department 2 days per week. We did not include in the study patients with diabetes duration less than 02 months, patients with diabetes secondary to endocrine, pancreatic or genetic diseases, and patients with gestational diabetes.

### Data collection

The study was conducted during the period from april to october 2017. Study participants had a face-to-face interview with the physician to collect data using a standardized questionnaire. The physician collected the following variables: age, educational level, profession, economic status, living conditions (living alone or with the family), type and duration of diabetes, smoking and alcohol habits, past history of hypertension, dyslipidemia, claudication, revascularization, lower limbs ischemia or confirmed arteritis, amputation, retinopathy, and foot ulceration or complicated diabetic foot.

Then, the physician proceeded to a clinical examination including measurement of blood pressure, waist circumference (WC), recording of the patient weight and height with calculation of body mass index (BMI), thorough feet examination searching for the presence of foot ulcers, gangrene, infection or other foot lesions, noting the hygiene, hyperkeratosis areas, toe web intertrigo, foot deformities (flat or hollow foot, clawed, straddled or hammer toes, hallux valgus and / or quintus varus and Charcot’s foot) and palpation of the pedic and posterior tibial arteries. Patients having active foot lesion were excluded from the study.

The PSN was assessed using the 5.07 (10 g) Semmes-Weinstein monofilament according to the technique suggested by IWGDF [3, 9]. The monofilament is first applied to the patient’s upper arm to demonstrate the sensation. Then the physician applies the filament perpendicular to the surface of the skin on three plantar sites: the apex of the hallux, and under the 1st and 5th metatarsal head, avoiding the areas of callus. She repeats the application three times at the same site. The examiner asks the patient, with the eyes closed, when and where he/she feels the pressure.

In the last step, the physician collected the results of the latest complementary exams: fasting glycaemia, glycated hemoglobin, plasma creatinine and creatinine clearance, ophthalmic examination, and results of explorations of lower extremities arteries if done

### Diagnostic criteria

PSN was present if at any site of application of the monofilament, at least two of the three responses were false [3, 9]. Screening of PAD was made on the basis of the patient’s medical history and clinical examination. PAD was present if there was an intermittent claudication and absence of pedic and posterior tibial pulses in the same side, or a history of vascular reconstruction or angioplasty or ischemic foot lesion (necrosis, gangrene) or documented PAD as confirmed by Doppler ultrasound examination of lower extremities arteries or by arteriography. Hyperkeratosis was diagnosed if presence of callus regarding hyperpression zones of the feet. Diabetes was considered well controlled if glycated hemoglobin was less than 7% and/or fasting glycaemia less than 140 mg/dl, it was considered poorly controlled if HbA1c more than 10% and/or fasting glycaemia more than 250 mg/dl and averagely controlled between these values.

We assessed the economic status on the basis of the patient’s head of family occupation and family income. We considered as low economic status if the patient’s head of family doesn’t work or has an unstable work, or the family income is less than 350 TND which was approximately the guaranteed minimum wage GMW. It’s considered high if patient’s head of family had a stable source of income and the family income is more than three times the GMW. Average economic status defines the patients with a family income between 1 and 3 times the GMW.

A patient was considered as having a poor psycho-social status if he was living alone and/or was alcoholic and/or had a serious psychiatric disease and/or had a poor body hygiene.

We classified patients according to the IWGDF system where four grades of increasing severity were identified [3]:

- Group 0: patients who had no PSN (low risk group)
- Group 1: patients who had isolated PSN
- Group 2: neuropathic patients who had foot deformity or PAD
- Group 3: prior foot ulcer or amputation (highest risk group)

### Statistical analysis

Statistical analysis was performed using SPSS version 21.0. Quantitative variables were presented as mean ± SD, while qualitative variables were presented as percentage. Comparison between risk groups was done using Pearson’s Chi^2^ test for qualitative data and T student’s test for quantitative data, *p* < 0.05 was statistically significant. In order to identify factors associated with a high risk lesion, we performed an univariate and then a multivariate analysis.

We looked for an association between the high risk groups and the following parameters: age, gender, geographic origin (rural or urban), school level, economic level, duration, equilibrium and type of diabetes, presence of diabetic retinopathy, creatinine clearence, psychosocial state, hypertension, hyperlipidemia, toe web intertrigo, hyperkeratosis, BMI and WC. For the univariate analysis, we compared the different parameters between the group 0 (low risk) and the groups 2 and 3 (high and very high lesion risk) according to IWGDF system.

These variables were tested for significance by a multivariate analysis to identify factors associated independently with high risk lesions. The co-variable adjustments were carried out by logistic regression with a significance of 0.2 and then the differences were considered significant if p <0.05 for a 95% confidence interval.

## Results

Among the 230 patients with diabetes examined, 10 patients were excluded because they presented during the examination an active foot lesion, 220 patients were available and then included in the study.

There were 114 females and 106 males, sex ratio 0.93. The mean age was 55.07 +/− 13.54 years (Mean age in males 52.88 +/− 15,14, females 57.11 +/− 11,55 years, *p* = 0.02). The distribution of the patients according to the school level was 39% illiterate, 38% primary, 19% secondary and 4% university level. The economic status was low in 10.90%, average in 87.72% and high in 1.36%. Among the 220 patients, 70.9% were living in an urban area and 29.1% in a rural area.

The habit of smoking was present in 42 patients all male, 39.62% of the male population study. Two patients were alcoholics and were also smokers. A poor psycho-social status was noted in 10.45% of the population’s study.

The mean BMI was 30.38 ± 5.47 kg/m2. An overweight (BMI 25 to 30 kg/m2) was observed in 37.27% and obesity BMI > 30 kg/m2) in 47.72% of the study population. An increased WC was seen in 96.33% of women (> 88 cm), 39.61% of men (> 102 cm), and in 65.90% of the total population.

The majority of the patients had type 2 diabetes (198 cases 90%) including 138 (62.27%) insulin-treated, 10% had type 1 diabetes. The mean duration of diabetes was 9.70 ± 6.59 years, it was less than 5 years in 36.57%. The diabetes was considered to be averagely controlled in the majority of cases (55.91%), well and poorly controlled in 15.91 and 28.18% of the cases successively. Hypertension and dyslipidemia were present in successively 38.18 and 34.54% of the patients.

Diabetic retinopathy was observed in 39.17% of the 194 patients examined, divided into 50% non-proliferative, 26.31% pre-proliferative and 23.68% proliferative diabetic retinopathy. The average creatinine clearance, calculated by the Cockcroft method, was 100.90 ± 40.19 ml/min.

Fifty-three patients had toe web intertrigo (24.09%), 76 patients (34.54%) had feet hyperkeratosis. A combination of toe web intertrigo and hyperkeratosis was found in 47 patients (21.36%).

Three patients (1.36%) had a history of amputation (one at the small toe and two trans-metatarsal). Five patients (2.27%) had a history of neuropathic plantar ulceration. Foot deformities were observed in 96 patients (43.63%): quintus varus (79.16%), hallux valgus (50%), overlapping toes (34.37%), flat foot (28.12%), claw toes (27.08%), hammer toes (15.62%), hollow foot (2.08%) and Charcot foot (1.04%). The prevalences of PSN and PAD were 23.63 and 36.82% respectively.

Therefore, the distribution of the patients according to the IWFDF classification was:

- Group 0: 160 cases (72.73%) including 42 cases (19.09%) with isolated PAD
- Group 1: 13 cases (5.90%)
- Group 2: 39 cases (17.73%)
- Group 3: 8 cases (3.64%)

After univariate analysis, we found that there was a significant association between risk severity and age, gender, BMI, WC, duration of diabetes, retinopathy, hyperkeratosis and school level. Compared to group 0 (low risk group), groups 2 and 3 (high risk group) were significantly older, were more often females, had android obesity, hyperkeratosis, retinopathy, longer diabetes duration and low school level (Table 1).

**Table 1:** Comparison between group 0 (low risk) and groups 2–3 (high and very high risk)

Variables^a^	Grade 0	Grade 2 and 3	P
	160 cases	47 cases	
Age (years)	53.50 +/− 14.36	58.04 +/− 9.88	0.044
Sex	Male: 86 (53.75%)	Male: 18 (38.29%)	0.045
	Female: 74 (46.25%)	Female: 29 (61.70%)	
Scholar level	Illetrate-Primary: 116 (72.5%)	Illetrate-Primary: 43 (91.5%)	0.008
	Secondary-University: 44 (27.5%)	Secondary-University: 4 (8.5%)	
BMI (kg/m2)	29.87 +/− 5.32	31.85 +/− 6.098	0.031
Waist circumferance	Normal: 55 (35.94%)	Normal: 8 (17.02%)	0.018
	Elevated: 98 (64.05%)	Elevated: 36 (76.59%)	
Diabetes duration (years)	8.45 +/− 6.46	11.96 +/− 7.19	0.002
Retinopathy	Present 43 (31.38%)	Present 26 (57.5%)	0.002
	Absent 94 (68.61%)	Absent 20 (42.55%)	
Hyperkeratosis	Present 47 (29.37%)	Present 25 (53.19%)	0.003
	Absent 113 (70.62%)	Absent 22 (46.80%)	

^a^ In this table are reported only significant associated factors in univariate analysis

*p* < 0.05 is considered to be statistically significant

There was no significant association between risk severity and economic level, rural/urban origin, type of diabetes, diabetes control, renal function (creatinin clearence), psychosocial state, hypertension, hyperlipidemia and toe web intertrigo.

Multivariate analysis identified 3 significant factors: the presence of retinopathy (OR = 2.529, 95% CI [1.131–5.655], *p* = 0.024), hyperkeratosis (OR = 2.658, 95% CI [1.222–5.783], *p* = 0.014) and school level (OR = 0.489, 95% CI [0.253–9.44], *p* = 0.033).

## Discussion

The present survey is the first done in Tunisia aiming to determine the prevalence of risk factors of foot ulcers and thus classify the patients to risk categories. Our study aimed also to identify factors associated with high risk foot ulcer in order to target future preventive measures. The clinical examination was made by the same physician reducing the bias of changing operator. Population study was randomly selected from patients attending the outpatient diabetology department. We found that 21.36% were high risk patients, and foot deformity was the most contributing factor. There was a clear trend between the increasing severity of the staging and the presence of retinopathy, hyperkeratosis and low school level. The limits of our study were the small size of the population, and some screening methods we used. Diagnosis of PAD was made on the basis of clinical examination and medical history. Non invasive vascular explorations were not available in our department. These tests have some limitations. Measurement of the ankle brachial index is the most widely used method to diagnose and quantify PAD. However, ankle pressures may be falsely elevated due to calcification of the arteries [9]. In these cases, toe pressure can be useful. The transcutaneous partial pressure in oxygen TcPO2 values are reduced in diabetic compared to non diabetic patients, more markedly in cases of neuroischemic foot compared to arterial controls without diabetes [10]. Measurement of TcPO2 is time consuming and expensive. Screening of PAD in the management of diabetic foot on large scale can be based on clinical findings as we done and as described in the international consensus on the diabetic foot [9]. The 2019 guidelines of the American Diabetes Association ADA confirm this attitude and recommend that patients with symptoms of claudication or decreased or absent pedal pulses should be referred for ankle brachial index and for further vascular assessment [11].

The presence of PSN was assessed in our study by the 5.07 Semmes Weinstein monofilament according to the IWGDF guidelines [9]. The 10-g monofilament test alone is useful for detecting advanced neuropathy and identifying patients at increased risk of ulceration and amputation [12–14]. In the studies that had used this test [12, 13], the location and number of sites tested and the definition of the PSN were different. The technique suggested by IWGDF, used in our study, has the advantage to be simple, rapid, not expensive and reproducible [15]. The ADA recommends annual 10-g monofilament testing with at least one other assessment: pinprick, temperature, vibration [11].

The prevalence of PSN in our study was 23.63%, less than the prevalence found by Malgrange et al. using the same methodology (27.1%) [16] Assaad-Khalil et al. [17] (29.3%) and Shahbazian et al. [18] (35%) using different sites for the monofilament in the first and a supplementary vibration test in the second. PSN plays a central role in the pathogenesis of foot ulcers. It leads to an insensitive and subsequently deformed foot with areas of elevated pressure when walking [2]. Measurement of foot pressure requires specialized materiel and is not recommended in routine management of diabetic foot. Attentive inspection of the patient’s feet is very important to detect hyperkeratosis and deformities [9]. In our study, the prevalence of foot deformities, 43.63%, was markedly high compared with other studies (20%) [16, 18] but similar to that found by Mugambi et al. in an african population (46%) [8]. These differences could be explained by patients’ age, diabetes duration, subjective criteria of diagnosis and also probably by ethnic differences. The presence of hyperkeratosis is highly predictive of future foot ulceration [19]. In our study, the prevalence of hyperkeratosis was 34.54%. This prevalence is rarely reported in the studies and is highly variable, 3% in the study of Shahbazian et al. [18], 45% in the study of Malgrange et al. [16]. Therefore, foot deformities and hyperkeratosis should be screened periodically in diabetic patients.

Several studies classified diabetic patients according to the IWGDF classification, we compared them to our study in Table 2. Group 0 was the less prevalent in the series of Vibha et al. [20] although it was a community based study. Group 3 was the less prevalent in our series, our center is not specialized in managing diabetic foot. Compared to the other studies, Group 1 was less prevalent in our study, PSN could be under-estimated, the 10 g Semmes-Weinstein monofilament test has been used in combination with other tests in the compared series [8, 18, 20].

**Table 2:** Prevalence of risk categories according to the IWGDF Risk Classification system in different studies

Author [reference] Country/year	Way of recrutement (number)	Group 0%	Group 1%	Group 2%	Group 3%
Malgrange [16] France/2003	In and outpatients (*n* = 555)	72.7	9.7	9.8	7.7
Mugambi [8] Kenya/2009	Outpatients (*n* = 218)	57	10	16	17
Shahbazian [18] Iran/2013	In and outpatients (*n* = 430)	65	17	11	7
Vibha [20] India/2018	Community based, home visit (*n* = 620)	48.2	31.4	11.9	8.5
Our series	Outpatients (*n* = 220)	72.72	5.9	17.73	3.63

Although the IWGDF classification system has been shown to predict diabetic foot complications [6], it may undervalue the impact of PAD and history of amputation. Modified versions of the IWGDF classification have been proposed, individualizing the group of isolated PAD as a risk group [16, 21], or separating the groups of ulceration history or amputation history [21]. In 2008, a modified IWGDF classification was recommended by the ADA and the American Association of Clinical Endocrinologists (AACE) [22]. Very recently, the IWGDF guidelines have been updated with a new classification that considers isolated PAD as a risk factor, and includs end stage renal disease as an aggravating factor [23]. Thus, category 0: No loss of protective sensation (LOPS) and no PAD, category 1: LOPS or PAD, category 2: LOPS + PAD, LOPS + foot deformity or PAD + foot deformity, category 3: LOPS or PAD, and one or more of the following: history of a foot ulcer, a lower-extremity amputation, and end-stage renal disease. This new classification needs to be evaluated by prospective studies.

Factors associated with high risk ulcer in our study were age, diabetes duration, female gender, elevated WC, BMI, retinopathy and hyperkeratosis; a high school level was associated with lower risk ulcer. Advancing age was found in some studies [16, 18, 20]. It may be explained by a longer diabetes duration, a risk factor of foot ulcer unanimely retrieved [6, 13, 16–18, 20, 24]. A longer diabetes duration is associated with development of PSN in many neuropathy prevalence studies [25, 26]. A female gender was associated with high risk ulcer in our study, in accordance with the results of Peters et al. [6]. No association with gender was retrieved in some studies evaluating this parameter [16, 18, 20]. A male gender was associated with a higher prevalence of diabetic foot ulcers or amputations in several studies [17, 27–29]. Our results may be explained by a more advanced age, a higher prevalence of elevated WC, and probably a lower school level in women in our study. Hyperkeratosis was identified as a risk factor in our study, in accordance with the results of Malgrange et al. [16], Assad-Khalil et al. [17] and Murray et al. [19]. Hyperkeratosis was considered as an indirect sign of elevated pressure foot areas and peripheral neuropathy [19].

Elevated BMI was associated in our study to a higher risk of foot ulcer. This association has not been retrieved in the studies of Malgrange et al. [16], Shahbazian et al. [15], Vibha et al. [20], and Wu et al. [23]. However, Sohn et al. found a linear association between BMI and foot ulceration at 1 and 5 years in a prospective study of patients less than 60 years with diabetes [30]. Gray et al. demonstrated that overweight was associated with lower extremity complications in the elderly with a HR 1.55 in women and 1.47 in men [31]. The increased risk associated with high BMIs can be explained by both biomechanical and pathophysiological mechanisms through increased pedal stress, and through aggravating diabetes and diabetic vascular complications [30]. Elevated WC was associated in our study to higher risk groups. A high WC was demonstrated to be a risk factor for diabetic neuropathy [32] or for diabetic foot ulcers [27, 28]. We could suggest the same mechanism for the elevated BMI.

A low school level was associated in our study with higher risk ulcer in accordance with the study of Vibha et al. [20] who identified also a low socioeconomic status as another risk factor, a result also demonstrated by Bansal et al. [25]. Socioeconomic status and school level are generally inter-related. We evaluated only economic status based on family income, difficult to assess only by the family head profession, and didn’t found a significant association. A good school level allows a better assimilation of the health care education and a better awareness.

Diabetic retinopathy has been identified as a risk factor of diabetic foot ulcers by several studies [13, 16–18, 24, 33] as shown in the current study. The presence of chronic diabetic foot ulcers was associated with more frequent and more advanced retinopathy [34]. Some studies showed higher levels of plasma uric acid and ceruloplasmin in diabetic foot patients with retinopathy [35, 36]. Ceruloplasmin was demonstrated to be an independent predictor for the progression of diabetic nephropathy in patients with type 2 diabetes [37] and is a potential biomarker of diabetic retinopathy [38]. Finally, changes of the microcirculation in patients with diabetes may lead to diabetic foot complications [39] as well as to retinopathy.

There was no significant association between risk severity and diabetes control. This observation is supported by some studies [20, 25] but not by others [6, 13, 18, 24]. We found no significant association with hypertension and hyperlipemia in accordance with previous studies [17, 20]. Hypertension was a foot ulcer risk factor in some studies [24, 33]. We did not found an association between creatinin clearence and foot ulcer risk grade. Nephropathy has been associated to foot ulcer risk [6, 18, 24]. Creatinin clearence is altered only in late stages of diabetic nephropathy, and may be affected by other causes.

Multivariate logistic regression revealed that retinopathy, hyperkeratosis and school level were significant factors of high risk foot ulcer. Hyperkeratosis and retinopathy were also identified by Leymarie et al. [7], with a more significant OR for retinopathy (OR = 4.20, CI95 [2.4–7.4] versus 2.52 in our study) and comparable OR for hyperkeratosis (OR = 2.30, CI95 [1.3–3.9] versus 2.65). In the study of Peters et al. [6], OR for RD was 8.9, CI95 [3.7–21.5]. In the Seattle prospective diabetic foot study, Boyko et al. [13] showed that poor vision was independently related to higher ulcer risk (RR 1.92, CI95 [1.39–2.64]), RR in patients with a normal vision who had laser photocoagulation was 1.79, CI95 [1.18–2.74]. Diabetes duration has been identified in different studies [7, 13, 17, 20] with OR = 2.2, CI95 [1.2–3.9]) [7]. and 2.40, CI95 [1.53–3.78] [20] for diabetes duration more than 10 years.

## Conclusion

This study was the first about risk assessment and classification of diabetic foot in this region and its findings can be useful in the prevention and management of diabetic foot. High risk patients are old female patients, with a long diabetes duration, complicated by retinopathy, having hyperkeratosis and android obesity. Patients with retinopathy or hyperkeratosis have approximately 2.5 folds higher risk to be high risk patients of foot ulcer, and patients with a secondary or university level education have one half lower risk to be in these categories. Our data highlight the value of a public health policy focusing on prevention by planning a regular screening for foot lesions and education of diabetic patients with a more attention to patients with low school level or having hyperkeratosis and retinopathy. Subsequently, we must focus on awareness of the patients. These measures should be applied to chronic disease outpatient structures in first line facilities, which are the first to deal with diabetic patients.

## Data Availability

The datasets generated and/or analysed during the current study are not publicly available, due to the possibility of identifying patients through the informations registred in the dataset but are available from the corresponding author on reasonable request.

## Abbreviations

ADA: The American Diabetes Association
BMI: Body mass index
GMW: Guaranteed minimum wage
IWGDF: The International Working Group on the Diabetic Foot
LOPS: Loss of protective sensation
PAD: Peripheral artery disease
PSN: Peripheral sensory neuropathy
TcPO2: The transcutaneous partial pressure in oxygen
WC: Waist circumference
